# Defining Auditory-Visual Objects: Behavioral Tests and Physiological Mechanisms

**DOI:** 10.1016/j.tins.2015.12.007

**Published:** 2016-02

**Authors:** Jennifer K. Bizley, Ross K. Maddox, Adrian K.C. Lee

**Affiliations:** 1 University College London (UCL) Ear Institute, 332 Gray's Inn Road, London, WC1X 8EE, UK; 2 Institute for Learning and Brain Sciences, University of Washington, 1715 NE Columbia Road, Portage Bay Building, Box 357988, Seattle, WA 98195, USA; 3 Department of Speech and Hearing Sciences, University of Washington, 1417 NE 42nd Street, Eagleson Hall, Box 354875, Seattle, WA 98105, USA

**Keywords:** multisensory, auditory cortex, psychophysics, neurophysiology, crossmodal, binding

## Abstract

Crossmodal integration is a term applicable to many phenomena in which one sensory modality influences task performance or perception in another sensory modality. We distinguish the term binding as one that should be reserved specifically for the process that underpins perceptual object formation. To unambiguously differentiate binding form other types of integration, behavioral and neural studies must investigate perception of a feature orthogonal to the features that link the auditory and visual stimuli. We argue that supporting true perceptual binding (as opposed to other processes such as decision-making) is one role for cross-sensory influences in early sensory cortex. These early multisensory interactions may therefore form a physiological substrate for the bottom-up grouping of auditory and visual stimuli into auditory-visual (AV) objects.

## Trends

Crossmodal integration and binding have been treated as synonymous in the literature, with no clear delineation between perceptual changes and other interactions such as decision-making.

Crossmodal binding is proposed as a distinct form of integration leading to multisensory object formation.

Multisensory stimuli are most beneficial in noisy situations, but few studies use stimulus competition to investigate the processes underpinning multisensory integration.

Evidence suggests that both visual and auditory attention is object-based – all features within an object are enhanced and there is a cost to attending features across versus within objects.

Multisensory interactions can be observed throughout the brain, including early sensory cortex.

The role of early sensory cortex in multisensory integration is unknown, but may underlie crossmodal binding.

## Introduction: What Is an AV Object?

**Figure 1:**
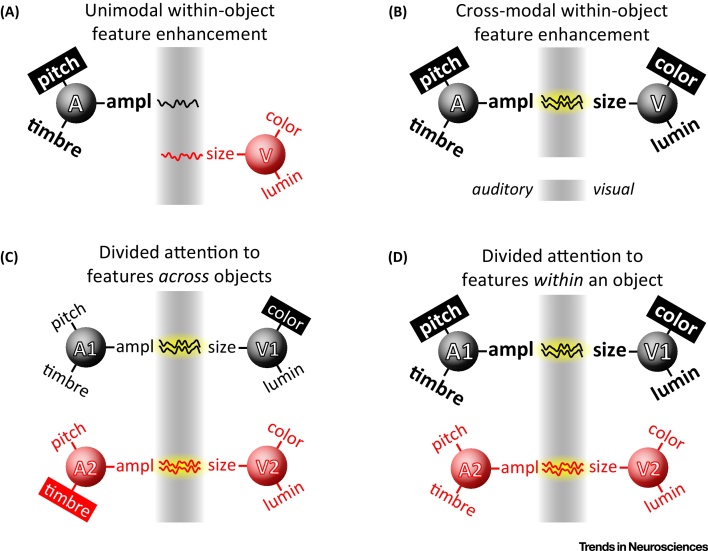
Binding of Features into Uni- and Crossmodal Objects Through Temporal Coherence. (A) When one attends to pitch (attended features shown with a highlighting box), all the other auditory features will be enhanced (depicted by an increased font size), but not features belonging to a separate visual object. (B) The amplitude of the sound is now comodulated with the size of the visual stimulus, and this temporal coherence (highlighted in yellow) enables these two sensory stimuli to bind into one object. We hypothesize that in this situation when one attends pitch, the ability to make color judgment would also be enhanced. This enhancement should also be bidirectional: when one makes a color judgment, there is enhancement in a pitch-related task [32]. (C) In a divided attention task, the cost of attending two features spanning across two objects should be more than when the two features belong to the same crossmodal object. (D) The perceptual cost can be measured behaviorally as a decrement in sensitivity of these features or an increase in reaction time compared to when both attended features are within the same crossmodal object. The fluctuating lines extending from “ampl” and “size” show those dynamic features’ time envelopes. Abbreviations: ampl, amplitude; lumin, luminance.

What we hear and see take strikingly different physical forms, and are necessarily encoded by different sensory receptor organs, but auditory and visual features are effortlessly bound together to create a coherent percept. Binding stimulus features from a common source is not only a problem across sensory systems – within sensory systems, parallel and independent perceptual feature extraction mean that stimulus features, such as pitch and space, must also be appropriately combined into a single perceptual object (Box 1, Figure 1A). The formation of cross-sensory objects is a problem synonymous with feature-binding in the visual system, or auditory scene analysis in the auditory system.

We define an AV object as ‘a perceptual construct which occurs when a constellation of stimulus features are bound within the brain’. Participating in a conversation at a crowded bar is assisted by pairing your friend's face and mouth movements with her voice; picking out the melody of the first violin in a string quartet is made easier by watching the player's bowing action. In each case what you see and hear are bound into a single crossmodal object. Conversely, trying to listen to one friend while watching another's face makes listening more difficult. These examples demonstrate two fundamental aspects of object-based attention 1, 2, 3, namely (i) attending to one feature of an object enhances the representation of the other features of the object (Figure 1B), and (ii) dividing attention between two features across two objects is costly compared to when attending to two features within the same object (Figure 1C,D). The purpose of this Opinion article is twofold. First, we wish to distinguish binding, which underlies crossmodal ‘objecthood’, from other mechanisms of **crossmodal integration** (see Glossary). In making this distinction we will argue that **crossmodal binding** can best be demonstrated by leveraging competition in tasks based on theories of object-based attention. Second, we propose that the purpose of early cross-sensory integration is to support binding.

## Section I. Behavioral Assays

### Binding as a Special Case of Multisensory Integration

**Figure 2:**
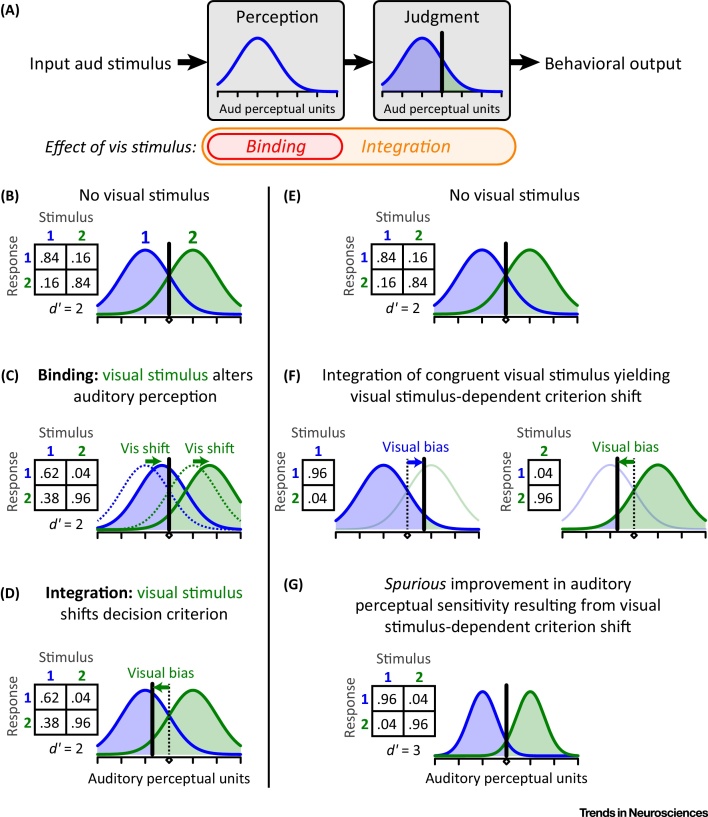
Interpreting Behavioral Results as Binding or Integration. (A) The processing underlying a psychophysical task involves perception of the auditory (aud) stimulus then judgment. Integration could happen at any stage. Binding implies a perceptual change. (B) An auditory task involving discriminating between auditory stimuli 1/2. Blue/green curves represent the conditional probability density function (PDF) for the perception of each stimulus. Perception below/above the decision criterion (vertical line) determines stimulus judgment as 1/2 (colored shadings). The table shows the probability of reporting stimulus 1/2 given 1/2 was presented, yielding *d′* of 2.0. (C,D) Different influences of a visual (vis) stimulus yielding identically biased behavioral responses, making the underlying mechanisms indistinguishable. In (C), visual stimulus binds with auditory stimulus, shifting auditory perception towards 2 (PDFs move rightwards), yielding reporting bias despite no criterion shift. In (D) there is no binding. The visual stimulus effects a leftwards criterion shift, again yielding bias towards reporting 2. Both (C) and (D) represent integration, but only (C) represents binding. (E–G) Stimulus-dependent decision bias (i.e., integration, but not binding) causing spurious measurements of improved perceptual sensitivity, (E) shows the task in the absence of a visual stimulus for reference. In (F) the visual stimulus in each trial is consistent with each auditory stimulus. Thus, for stimulus 1 (left), the visual stimulus pushes the criterion to the right, biasing responses towards stimulus 1 (correct response), and similarly for stimulus 2 (right). There is no change (neither shift nor expansion/contraction) in either PDF (G). These responses yield *d′* of 3.0 and the erroneous conclusion that perceptual sensitivity has increased. However, no binding has occurred.

There are a multitude of ways in which stimuli in one sensory modality can influence or perturb the behavioral response to stimuli in another modality 4, 5. We conceptually describe two stages of interaction (Figure 2A): first, the features of an incoming sound are perceived in a manner related to its physical value (e.g., a physical intensity is coded as loudness according to a probability distribution) and, second, this percept is subsequently used as the basis for a judgment about the sound. The term crossmodal (or multisensory) integration applies to any instance in which one sensory modality influences the judgment of stimuli in an other, and could therefore occur at either of these two stages. One example of crossmodal integration would be weighting information from different sensory modalities by their reliability to reach a decision 6, 7, 8.

Binding, we argue, is a specific concept that should be reserved for crossmodal linking of perceptual features resulting in a unified AV object (i.e., the change happens in the first stage of Figure 2A). Binding is a form of integration; however, integrating information at the decision stage, for example, is not a form of binding. We further argue that binding relies upon consistency between the modalities – in particular their temporal coherence [9]. Binding can theoretically be built on other consistencies, such as agreement in auditory and visual spatial location, or phoneme–viseme relationships (e.g., between an auditory /u/ vowel and an image of protruded lips); however, when such features are dynamic and coherent, the binding should be substantially strengthened. It is also worth noting that, for a dynamic stimulus, the only way for features to be consistent as they change in time is for them to be temporally coherent. Furthermore, temporal coherence allows disparate cross-sensory features to be bound, such as pitch and color (which have no presumed natural connection), thereby forming a coherent multisensory perceptual object. We see binding as a largely perceptual, rather than cognitive, process – and therefore distinct from the integration of information at the decision-making level. Importantly, as discussed in Section II, the processing stage that is affected by a multisensory stimulus carries implications about where in the brain the influence may be occurring.

Distinguishing binding from other forms of integration experimentally is non-trivial; most previous studies, while demonstrating a diverse range of integration effects, fall short of unambiguously showing binding due to a change in perception. Figure 2B–G demonstrates several ways in which behavioral results that may seem to show binding can actually be the result of integration at a later stage of processing. For example, given a certain pattern of behavioral responses for an auditory-only stimulus (Figure 2B), observing a bias in discrimination with a simultaneous visual stimulus can result from a shift in perception (which would be binding, Figure 2C) or from a shift in the decision criterion (which is integration, but is not binding, Figure 2D). Importantly, the behavioral readouts of these are identical, rendering the underlying processing changes indistinguishable. A change in sensitivity, seemingly free of bias, does not necessarily show binding either, as demonstrated in Figure 2E–G. If a visual stimulus changes in a way that is consistent with changes in the auditory stimulus, it can lead to a variable decision criterion that biases towards the correct behavioral response in a stimulus-dependent manner. The result might be interpreted as a measured improvement in sensitivity, with zero bias, that belies the underlying mechanism. There may actually be a perceptual change resulting from binding, but, in these situations – namely where the visual stimulus could reasonably shift the decision criterion for the auditory task – it is not possible to know. In fact, a variable decision criterion is a direct violation of a central axiom of the decision model used in signal detection theory: the criterion value must remain constant throughout an experiment for each perceptual judgment 10, 11. Thus, applying signal detection theory as in Figure 2G where the criterion may in fact be changing (Figure 2F) is flawed.

### Behavioral Tests for Identifying Binding: The Necessity of Assessing Stimulus Features Orthogonal to Those Leading to Binding

The question then becomes: how do we demonstrate binding experimentally? We believe that there is an essential experimental element to achieving this empirically: behavioral measures must be made on a stimulus feature orthogonal to the features that create the binding. In other words, none of the features that are intended to bind should be the dimension on which subjects report some type of perceptual judgment. A crossmodal feature orthogonal to that being tested will not influence the decision criterion. Thus, any measured changes in behavior can be assumed to result from changes in perception. This approach has been successfully used in unimodal studies, for example to objectively measure auditory object formation [12].

How could an **orthogonal stimulus feature** affect perception? If stimulus features are being bound to form a perceptual object then, consistent with object-based theories of attention 1, 2, 3, 13, all of the features of that object should subsequently be enhanced. These assumptions are easily extendable to the case of crossmodal objects. Furthermore, by making a judgment on a feature orthogonal to the features that lead to binding, we remove the possibility of crossmodal influence through simple decision biasing.

A wide variety of experimental paradigms have been used to investigate crossmodal interactions. Of these, illusions have gained particular traction because they provide insight into the obligatory mechanisms for resolving conflicting multisensory information. Reports of illusory percepts have been held up as demonstrating multisensory integration and crossmodal binding, but without consideration of their differences. We use three commonly investigated illusions to show how the bulk of these studies demonstrate multisensory integration and are consistent with – but do not conclusively indicate – binding of features into AV objects at the perceptual level. These examples also highlight the need for tests based on orthogonal features.

The first example is the **ventriloquist illusion** 7, 14. Here the location of a sound-source can be ‘captured’ by a visual stimulus, for example, the voice of the ventriloquist appears to come from her puppet's moving mouth rather than from her own (stationary) mouth. This illusion is compelling; intuitively, we have the impression that the voice and mouth have been ‘combined’ to form an object. However, does this illusion necessarily tell us that the brain has bound the auditory and visual signals? Observers have been shown to weigh their estimate of location according to the reliability of the signals in each modality in a manner consistent with Bayesian decision-making [7]. This finding suggests that independent estimates are made for each modality at a later decision-making stage that combines information across sensory modalities, and would therefore be consistent with our definition of crossmodal integration, rather than crossmodal binding. If observers are asked to localize both the auditory and visual source separately, then the location of the sound is much less biased than when observers treat the two signals as a single source, suggesting independent perceptual estimates are maintained 15, 16, 17. Moreover top-down factors such as emotional valence or reward expectation can alter the magnitude of the ventriloquism effect observed 16, 17. Such top-down mediation of the effect suggests that it is, at least in part, attributable to changes in judgment rather than perception.

A second commonly used illusion is the **McGurk effect**, in which a video of a mouth movement affects the auditory phoneme that listeners report hearing [18], whereby the percept is neither of the veridical unisensory percepts but is instead a third one. This illusion is also influenced by higher-level contextual effects 19, 20 as well as visual attention [21]. Even in these cases the illusory percept could represent a bias in the consonant perception continuum, influenced by both auditory and visual stimuli. Such processes, in which stimuli in each modality are coded independently, and then a judgment is made by considering the information provided by each, are consistent with the results discussed above for the ventriloquist illusion [7], as well as other illusory [22] and non-illusory multisensory behaviors [6]. Furthermore, uncertainty in visual speech signals can also potentially explain some of the observations in McGurk paradigms [23], making it unclear at what stage the integration is occurring. One study that does test an orthogonal feature [temporal (a)synchrony as opposed to phoneme identity] finds that subjects are more sensitive to asynchronies in illusory audiovisual syllables than in congruent ones, suggesting that the former are not integrated as strongly, further casting doubt on binding as the sole explanation for the illusion [24].

A third commonly explored illusion is the **sound-induced flash illusion** (SIFI) [25]. The SIFI is an illusion in which brief visual and auditory stimuli are presented rapidly, and the number of auditory stimuli influences the reported number of visual stimuli. Signal-detection theory analysis has demonstrated that illusory flashes are accompanied by measured changes in sensitivity (and not only bias) 26, 27, suggesting that this illusion is due in part to a change in perception. However, illusory flashes are not perceived in the same way as real flashes: when offered a third ‘not-one, not-two’ option many subjects choose it [28]. While most experiments utilizing the SIFI do not fulfill our proposed criteria of testing for AV object formation, by using a stimulus feature orthogonal to the one being bound, a few do. One recent study asked subjects to not only count the number of flashes but also describe their color (an orthogonal feature dimension) [29], and a second tested contrast perception in addition to the number of events [27] and found that the effect is likely explained by both a perceptual change as well as a criterion shift.

Most studies of illusions equate changes in stimulus judgments with truly altered perception resulting from binding. However, with a few exceptions 24, 27, 29, all three illusion paradigms suffer from the problems indicated in Figure 2 – the dimension that links the auditory and visual stimuli is not orthogonal to the stimulus dimension being judged, making it impossible to tell binding apart from other forms of multisensory integration. In the ventriloquist illusion, visual space influences auditory spatial judgments; in the McGurk effect, a visual speech cue influences the phoneme reported; in the SIFI, the number of auditory events influences the number of visual events reported. The ambiguity inherent in interpreting such results underscores the need for testing features orthogonal to the crossmodal influence if one wishes to conclusively demonstrate crossmodal binding.

### Behavioral Tests for Identifying Binding: The Case for Stimulus Competition

In most laboratory situations, experimenters do not impose competition in their experimental design. We argue that, by introducing an element of stimulus competition, we can draw upon the object-based attention literature to generate specific and testable predictions about the processing advantages conferred upon the features associated with a single AV object. We further argue that introducing stimulus competition provides a more naturalistic and taxing situation that increases the perceptual benefit offered by crossmodal binding, making binding easier to detect. Competition has been proposed as similarly important for studying multisensory attentional processing [8].

The importance of stimulus competition can be demonstrated by comparing the outcomes of studies testing the influence of space on the SIFI. Those studies showed that the probability of an illusory percept was uninfluenced by the degree of spatial separation between auditory and visual stimuli [30], and that visual sensitivity was the key determinant of the probability of perceiving a SIFI, and not audiovisual spatial proximity [26]. However, when two spatially separated stimulus streams were placed in competition, and subjects were instructed to direct their spatial attention to only one of these streams, spatial factors became apparent [31].

A crossmodal object should be more salient than a unimodal one, which should provide a processing benefit, especially in the case of competing stimuli. For example, a recent study engaged observers in a selective attention task which required that they report brief pitch or timbre deviants in one of two ongoing independently amplitude-modulated sound-streams [32]. Observers also attended a radius-modulated disk that changed coherently with the amplitude of either the target stream or the masker stream, and were asked to report occasional brief changes in color. Performance was better when the visual stimulus was temporally coherent with the target auditory stream than when it was coherent with the masker stream. Because the modulations of the visual stimulus offered no information as to the timing of the target auditory deviants, the authors suggested a conceptual model proposing that the temporally-coherent auditory and visual streams formed an AV object whose properties were subsequently enhanced. Thus when the auditory target stream and the visual stimulus were bound into a single object, performance was improved because observers were no longer required to divide their attention across two sensory objects. Drawing on theories of object-based attention 1, 8, this model [32] leads to testable behavioral predictions. For example, while this study showed visual enhancement of auditory perception, we expect an equivalent auditory enhancement of visual perception [32].

In this section we have delineated the difference between binding leading to crossmodal object formation from broader integration, and suggest ways to determine unambiguously the existence of binding. We now discuss the differing neural processing that underlies these distinct ideas and offer guidelines for neural experiments to complement the behavioral approaches discussed above.

## Section II. Neural Basis

**Figure 3:**
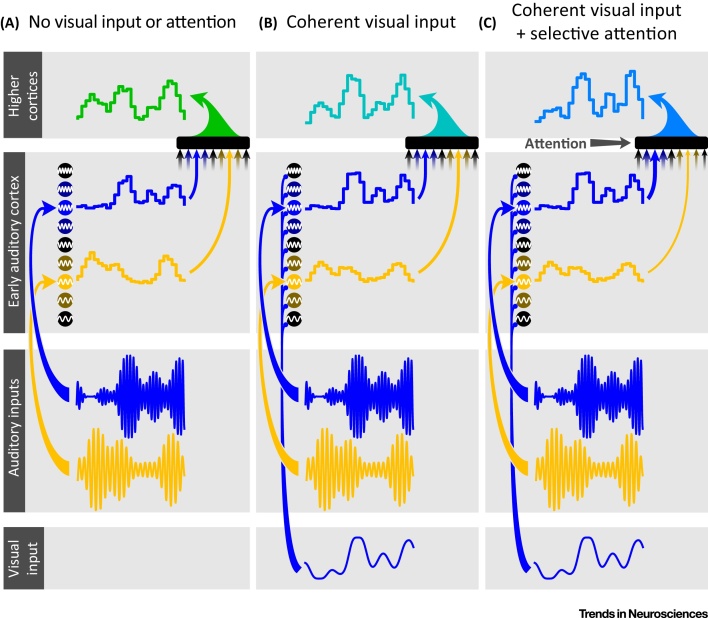
The Effect of a Coherent Visual Stimulus and Selective Attention on the Neural Representation of Competing Auditory Streams. (A) Starting at the bottom of the figure, two amplitude-modulated tones are the auditory inputs. These inputs are processed by neurons arranged in a tonotopic array such as those found in early auditory cortex (with white sinusoids denoting best frequencies), generating two peristimulus time histograms (PSTH) at each of the respective outputs. The two streams are equally salient (i.e., equal overall spike rates, but different temporal patterns). These two streams are combined at higher levels of processing with equal weighting. The color of the PSTH in the higher cortices reflects its similarity to each of the two input waveforms (blue and yellow). (B) The same as (A) but with a modulated visual stimulus that is coherent with the higher-frequency auditory input (blue). The influence of the blue modulation time-course (which could be achieved through spiking or subthreshold visual inputs) enhances the response to the blue (higher-frequency) waveform and reduces the response to the yellow (lower-frequency) waveform. This results in the response in higher cortices more resembling the blue waveform than the yellow. (C) The same as (B) but with the added effect of selective attention to the higher-frequency band resulting in the final neural readout resembling even more the original blue input.

What mechanism might allow temporally-coherent auditory and visual information to be bound? In Figure 2 we drew a distinction between multisensory integration at the level of perception (decision-making remains unchanged, but a crossmodal input alters the stimulus coding and consequently perception) and at the level of decision-making (the stimulus coding within a modality is unchanged but information in another modality changes the way in which the stimulus is interpreted). Specifically, we highlighted binding as a perceptual effect rather than one that was caused by interactions at the level of decision-making (Figure 2A). Multisensory interactions are found in a multitude of cortical and subcortical locations [4], and it seems likely that different anatomical loci might perform different types of multisensory integration. We propose here that multisensory integration in early sensory cortex provides the neurophysiological substrate for crossmodal binding. We extend the conceptual model described above to a neurophysiological one in which a visual stimulus can modulate the activity of auditory cortical neurons via direct feedforward or lateral visual inputs into auditory cortex, providing a mechanism through which auditory and visual stimuli can be bound together, enhancing their representations (Figure 3). Because neural activity in early auditory cortex is closely tied to perception 33, 34, integrating visual information early would facilitate genuine perceptual shifts. We argue that visual inputs to auditory cortex might act to enhance the activity of neurons that represent a sound-source that is coherent with a visual stimulus, and that this enhanced neural subpopulation could form the substrate on which top-down connections mediating selective attention further hone neural processing.

As in the behavioral studies mentioned above, we argue that the use of a competing stimulus stream will be instrumental in elucidating the neural mechanisms underlying binding and AV object formation. Moreover, the use of stimulus competition offers the potential to test the hypothesis that visual stimulus-induced neural correlates of binding occur independently of selective attention – something that is impossible to test with any form of behavioral paradigm. It has been observed that a second sound-source can radically change the response properties of neurons in auditory cortex even in the absence of selective attention 35, 36. Whereas single neurons display broad sensitivity to sound-sources presented in isolation, when two sources are presented in competition neural selectivity is greatly enhanced, resulting in discrete populations of neurons each representing one of the two competing sources 35, 36. We predict that a visual stimulus would selectively enhance (or suppress) neural populations based on their temporal (in)coherence.

Consistent with the variety of forms that multisensory integration can take, crossmodal interactions can be observed at many processing stages in the brain from sensory thalamus 37, 38 to prefrontal cortex 39, 40, 41. However, we propose that cross-sensory inputs to early sensory cortex play a key role in binding and thus AV object formation, while multisensory processing at later sites predominantly supports other forms of multisensory integration such as decision-making.

### Early Cross-Sensory Interactions: A Mechanism for Binding Auditory and Visual Information?

We suggest that the pattern of anatomical innervation and physiological response properties in early sensory cortex make them ideally suited to supporting the binding of auditory and visual stimulus features. The incidence of early multisensory interactions has been demonstrated in rodents 42, 43, 44, carnivores [45], non-human primates 46, 47, 48, and humans 49, 50, 51, 52. Anatomical studies reveal a plethora of potential bottom-up and lateral visual inputs, including primary visual cortex, secondary visual cortex, and multisensory thalamus 43, 45, 47, 53, 54, and functional evidence is consistent with a role for visual inputs in feedforward processing 45, 55, 56. Direct connections from auditory to visual cortex have been shown to modulate both spiking activity in visual cortex and visual perception, demonstrating that direct cortico-cortical connections can mediate multisensory integration [44]. Despite this evidence, the role of such early cross-sensory projections remains elusive – likely in part because of the relative scarcity of invasive neurophysiological studies in behaving animals performing multisensory tasks.

AV interactions within auditory cortex can be facilitative or suppressive, and may be visible as a spiking response 45, 57, 58, 59, an evoked local field potential response 58, 60, or as an entrainment of rhythmic activity across cortical areas 61, 62. The relative timing of auditory and visual signals can influence the nature of multisensory interactions such that the same neuron can show facilitation or inhibition depending on the AV (a)synchrony 45, 59, 63, 64. Spatial coincidence also determines the nature of multisensory interactions in both subcortical and cortical structures 57, 63, 65. Thus the properties of multisensory interactions in early sensory cortex are known to be dependent on the temporal coherence necessary for binding 58, 60. Most invasive studies of multisensory integration in sensory cortex have relied upon artificial stimuli presented as brief transient bursts, while parameters such as their onset timing or spatial location have been manipulated. Studies with more naturalistic stimuli suggest that temporally-dynamic visual stimuli can modulate the local field potential and spiking activity in a manner that improves the reliability of auditory cortical responses [66]. Stimulus-evoked deflections of the field potential could potentially modulate the firing rates of cortical neurons because activity from coincident sound arriving at high-excitability phases of the field potential 48, 58, 66 will be enhanced, while activity arriving at non-coincident times is likely to fall in low-excitability phases, thus decreasing the overall response to the acoustic stimulus. Spiking inputs (more common in secondary areas) could modulate activity in the same way, but provide more robust modulation (Figure 3). For a recent review of the mechanisms that might support these effects see [67].

Evidence consistent with the idea that interactions between sensory cortices play a key role in binding auditory and visual stimulus features is provided by human neuroimaging studies. The SIFI is thought to be mediated via early sensory cortex [68]. Modulation of the evoked EEG response to McGurk stimuli correlates with perception of the illusory syllables [69] and, although there is no direct mapping of EEG topography to underlying neural sources, modulation of the earliest components is consistent with visual stimuli eliciting changes occurring in auditory cortex. A concurrent visual stimulus can enhance the representation of a sound-source in human auditory cortex both for single sound-sources [41] and when listeners are faced with two competing talkers [70]. In the first instance such an effect could result simply from a multisensory stimulus being more salient, and would not necessarily indicate binding nor confer any perceptual advantages. In the second case, however, the representation of the coherent sound-source is enhanced over that of the competing stream; this finding cannot be explained by a general effect of arousal and is therefore likely to be indicative of binding.

If early sensory areas mediate binding then what are the roles of multisensory responses in other brain regions? Multisensory interactions are not exclusive to early sensory cortex and occur throughout the brain in various forms. For example, neurons in prefrontal cortex are sensitive to multimodal mismatch 40, 71, 72. Tasks requiring a level of semantic processing highlight areas, including the superior temporal sulcus (STS) and intraparietal sulcus (IPS), where the size of multisensory enhancement observed predicts the behavioral advantage an individual shows for multisensory over unisensory object classification 41, 73. Feedforward and lateral connections in early sensory cortex are also complemented by feedback connections from higher brain areas such as the STS and IPS 60, 74, 75, 76, and one role for these connections is proposed to be in mediating predictive coding [77] or object recognition [76].

### Physiological Tests for Binding

We have identified several features that are consistent with the hypothesis that multisensory integration in early auditory cortex plays a key role in binding and hence AV object formation. We suggest that the following observations would demonstrate a role for early cross-sensory interactions in the formation of an AV object. (i) In the presence of two competing auditory stimuli a visual stimulus that is temporally coherent with one stream should boost the representation of the subset of neurons that are driven by that auditory stream. This could occur by facilitating the responses of the neurons responding to the coherent sound-stream and/or suppressing the responses of neurons responding to the temporally-incoherent stream, and would require invasive neurophysiological recordings to disambiguate these two possibilities. (ii) These physiological effects should be observed in the absence of attention, but we predict that selective attention to the coherent AV source would further amplify them. (iii) A crucial test for a neural correlate of binding is an enhancement of all of the features that a neuron represents and not simply those that are temporally coherent with the visual stimulus. For example, a neuron in which visual and auditory information are bound by coherent changes in sound intensity and visual luminance should be better able to represent a change in other features of the sound-source than when the luminance and intensity vary independently. (iv) Finally, eliciting a behavioral deficit in a task that requires crossmodal binding by selectively silencing inputs from visual cortex to auditory cortex would provide a causal demonstration of the importance of interactions between sensory cortices in forming AV objects. While some studies looking at macro-scale signals with human imaging methods have provided empirical evidence in favor of the first of these proposals (e.g., [70], addressing point ii above) the remaining points remain unaddressed.

## Concluding Remarks

We have provided a functional definition of an AV object as a perceptual construct which occurs when a constellation of stimulus features are bound within the brain. Further, we identified binding as a specific case of multisensory integration in which stimulus features are perceptually grouped across modalities, leading to the formation of crossmodal objects. We argue that the formation of an AV object can be determined by demonstrating a crossmodal influence on a feature dimension orthogonal to the one that promotes binding. We further believe that stimulus competition is important because binding is most beneficial under naturalistic listening situations with multiple sound-sources, and such situations provide a way of investigating binding based on theories of object-based attention. Finally, we propose that feedforward or lateral cross-sensory connections in early sensory cortex facilitate binding.

Outstanding Questions *Bidirectionality.* We have focused on visual influences on auditory scene analysis and within auditory cortex. Our behavioral predictions should be bidirectional across modalities. Does attending to a sound also enhance visual judgments in a bound crossmodal object?

*Competing Visual Stimuli.* We have focused on the role of a single visual stimulus on an auditory mixture – but what about multiple competing visual stimuli? Do the same rules apply and how do they trade off against each other?

*Interaction with Attention*. As has recently been highlighted for multisensory attentional processing [8], stimulus competition is likely to be crucial to revealing such interactions: in the instance of single auditory or visual event streams, each is highly salient and they are likely to be automatically bound. How does bottom-up sensory integration interact with top-down attention both behaviorally and at the cellular level?

*Active Sensing.* What role do eye and head movements play in forming AV objects in a realistic multisensory environment?

*Neural Underpinnings of Audiovisual Binding.* Which (anatomical) inputs might shape neuronal response modulation in auditory cortex? How are diverse latencies compensated for? Do effects ‘build up’?

## Box 1 Forming Auditory and Visual Objects

Auditory and visual objects share particular properties: both have linked features that change over time and perceptually group together the acoustic or visual features that come from a common source [78]. The process of forming objects is often described as scene analysis in audition [79] and image segmentation [80] in vision. In both instances a continuous sensory input is represented across an array of sensory receptors in the cochlea or retina, and this input must be appropriately segmented into components 78, 80. Segmenting a visual image is computationally difficult because objects can occlude one another, although arguably the challenge faced by the auditory system is greater because a single sound-source can elicit a complex, discontinuous pattern of activity at the cochlea and multiple sound-sources may elicit overlapping or interdigitating patterns of activity.

Objects must therefore be inferred from low-level cues: for example, in the auditory system, acoustic cues such as inter-aural timing and level differences, used for localizing sound in the horizontal plane, and harmonicity lead to perceptual features (space and pitch respectively) that define an object and enable grouping of sound-elements from the mixture of sounds that form an acoustic scene [79]. Similarly, in the visual domain, objects are defined by perceptual features such as location, color, and shape that allow them to be separated from other objects in the environment 81, 82.

For sensory objects, the ability to group stimulus features enables the separation of the object from the features of competing stimuli that comprise the whole sensory representation [83]. In both vision and audition the formation of a perceptual object allows a level of abstraction that facilitates perceptual invariance and subsequent object recognition 78, 83, 84. For both auditory and visual objects, observers can deploy top-down mechanisms to selectively attend to an object of interest by focusing on a particular stimulus feature – such as color or pitch 3, 85. Selective attention can also be engaged by automatic, bottom-up mechanisms driven by salient attributes of a visual [2] or auditory [3] scene. Evidence suggests that both visual [1] and auditory [86] attention are object based as demonstrated by changes in the continuity of a task-irrelevant feature having an influence on behavior.

## Glossary

Crossmodal binding: a specific form of crossmodal integration wherein perceptual features are grouped into a unified crossmodal object.
Crossmodal integration: any process in which information across sensory modalities is combined to make a perceptual judgment.
McGurk effect: an illusion whereby mismatched visual mouth movements and auditory phonemes result in a merged percept. For example, a visual /ga/ and an auditory /ba/ are perceived as a /da/.
Orthogonal stimulus feature: perceptual dimensions that do not depend on one another. For example, the pitch of a sound can vary independently from its perceived location.
Sound-induced flash illusion (SIFI): a phenomenon in which the number of visual flashes reported is influenced by the number of rapidly presented auditory stimuli.
Ventriloquist illusion: an illusion whereby the spatial location of a sound-source is ‘captured’ by a visual stimulus. For example, we perceive an actor's voice as originating from the image of their mouth on a screen rather than from the loudspeakers around the cinema.
